# Metabolic tumour and nodal response to neoadjuvant chemotherapy on FDG PET-CT as a predictor of pathological response and survival in patients with oesophageal adenocarcinoma

**DOI:** 10.1007/s00330-023-09482-7

**Published:** 2023-03-15

**Authors:** Jonathan L. Moore, Manil Subesinghe, Aida Santaolalla, Michael Green, Harriet Deere, Mieke Van Hemelrijck, Jesper Lagergren, Sugama Chicklore, Nick Maisey, James A. Gossage, Mark Kelly, Cara R. Baker, Andrew R. Davies, On behalf of the Guy’s and St Thomas’ Oesophago-gastric Research Group, A. Jacques, N. Griffin, V. Goh, S. Ngan, K. Owczarczyk, A. Sita-Lumsden, A. Qureshi, F. Chang, U. Mahadeva, B. Gill-Barman, S. George, M. Ong, J. Waters, M. Cominos, T. Sevitt, O. Hynes, G. Tham, J. M. Dunn, S. S. Zeki

**Affiliations:** 1 Department of Upper Gastrointestinal and General Surgery, Guy’s & St Thomas’ Hospital, East Wing Link Corridor, London, SE1 7EH UK; 2 School of Cancer and Pharmaceutical Sciences, King’s College London, London, UK; 3 King’s College London & Guy’s & St Thomas’ PET Centre, London, UK; 4 Department of Cancer Imaging, School of Biomedical Engineering and Imaging Sciences, King’s College London, London, UK; 5 Translational Oncology and Urology Research (TOUR), School of Cancer and Pharmaceutical Sciences, King’s College, London, UK; 6 Department of Histopathology, Guy’s & St Thomas’ Hospital, London, UK; 7 Upper Gastrointestinal Surgery, Department of Molecular Medicine and Surgery, Karolinska Institutet, Stockholm, Sweden; 8 Department of Medical Oncology, Guy’s & St Thomas’ Hospital, London, UK

**Keywords:** Oesophageal neoplasms, Positron emission tomography-computed tomography, Neoadjuvant therapy, Pathology, Surgical, Surgical oncology

## Abstract

**Objectives:**

2-deoxy-2[^18^F]Fluoro-d-glucose (FDG) PET-CT has an emerging role in assessing response to neoadjuvant therapy in oesophageal cancer. This study evaluated FDG PET-CT in predicting pathological tumour response (pTR), pathological nodal response (pNR) and survival.

**Methods:**

Cohort study of 75 patients with oesophageal or oesophago-gastric junction (GOJ) adenocarcinoma treated with neoadjuvant chemotherapy then surgery at Guy’s and St Thomas’ NHS Foundation Trust, London (2017–2020). Standardised uptake value (SUV) metrics on pre- and post-treatment FDG PET-CT in the primary tumour (mTR) and loco-regional lymph nodes (mNR) were derived. Optimum SUV_max_ thresholds for predicting pathological response were identified using receiver operating characteristic analysis. Predictive accuracy was compared to PERCIST (30% SUV_max_ reduction) and MUNICON (35%) criteria. Survival was assessed using Cox regression.

**Results:**

Optimum tumour SUV_max_ decrease for predicting pTR was 51.2%. A 50% cut-off predicted pTR with 73.5% sensitivity, 69.2% specificity and greater accuracy than PERCIST or MUNICON (area under the curve [AUC] 0.714, PERCIST 0.631, MUNICON 0.659). Using a 30% SUV_max_ threshold, mNR predicted pNR with high sensitivity but low specificity (AUC 0.749, sensitivity 92.6%, specificity 57.1%, *p* = *0.010*). pTR, mTR, pNR and mNR were independent predictive factors for survival (pTR hazard ratio [HR] 0.10 95% confidence interval [CI] 0.03–0.34; mTR HR 0.17 95% CI 0.06–0.48; pNR HR 0.17 95% CI 0.06–0.54; mNR HR 0.13 95% CI 0.02–0.66).

**Conclusions:**

Metabolic tumour and nodal response predicted pTR and pNR, respectively, in patients with oesophageal or GOJ adenocarcinoma. However, currently utilised response criteria may not be optimal. pTR, mTR, pNR and mNR were independent predictors of survival.

**Key Points:**

*• FDG PET-CT has an emerging role in evaluating response to neoadjuvant therapy in patients with oesophageal cancer.*

*• Prospective cohort study demonstrated that metabolic response in the primary tumour and lymph nodes was predictive of pathological response in a cohort of patients with adenocarcinoma of the oesophagus or oesophago-gastric junction treated with neoadjuvant chemotherapy followed by surgical resection.*

*• Patients who demonstrated a response to neoadjuvant chemotherapy in the primary tumour or lymph nodes on FDG PET-CT demonstrated better survival and reduced rates of tumour recurrence.*

**Supplementary Information:**

The online version contains supplementary material available at 10.1007/s00330-023-09482-7.

## Introduction

2-Deoxy-2[^18^F]fluoro-d-glucose positron emission tomography-computed tomography (FDG PET-CT) is routinely used for staging of oesophageal cancer and has an emerging role in evaluating response to neoadjuvant therapy [1, 2]. A reduction in FDG avidity of the primary tumour and loco-regional lymph nodes (LN) following neoadjuvant chemotherapy has been shown to predict pathological response and survival [1–4]. However, no studies have evaluated the prognostic significance of metabolic parameters derived on FDG PET-CT with respect to pathological response in LNs, but have instead used primary tumour response or pathological nodal stage as a surrogate [5]. This is despite emerging evidence of a discrepancy, in up to 25% of patients, between the response in the primary tumour compared with that in LNs [1, 6, 7]. One hypothesis is that this discrepancy may be due to variations in chemo-sensitivity of different clonal populations of tumour cells [6, 8].

Change in primary tumour maximum standardised uptake value (SUV_max_) is the most widely used FDG PET-CT parameter to define metabolic response to treatment with various thresholds described. The most commonly utilised are a 35% reduction in SUV_max_ as adopted by the MUNICON trial [9] and PET Response Criteria in Solid Tumours (PERCIST) which recommends a 30% reduction to define response [10]. However, there is no consensus regarding the optimal classification; the MUNICON threshold was derived from only 40 patients [11] and PERCIST is not tumour specific.

The primary aim of this study was to evaluate the ability of FDG PET-CT to predict pathological response in the primary tumour and LNs for patients with adenocarcinoma of the oesophagus or oesophago-gastric junction undergoing neoadjuvant chemotherapy prior to surgical resection. Secondary aims were to assess the prognostic effect of metabolic and pathological response on survival and to evaluate the predictive ability of currently utilised response classifications.

## Methods

### Design

This was a cohort study based on an ethically approved, prospectively maintained database of consecutive oesophago-gastric resections performed at Guy’s and St Thomas’ NHS Foundation Trust, London, UK. The present study included patients treated with neoadjuvant chemotherapy who underwent oesophago-gastrectomy with curative intent for histologically confirmed adenocarcinoma of the oesophagus or oesophago-gastric junction between January 2017 and December 2020. All patients had locally advanced tumours, i.e. cT2 or higher, positive LNs or both. Tumours were staged using the American Joint Committee on Cancer TNM version 8 [12]. Tumours of the oesophago-gastric junction were categorised using the Siewert classification [13]. The primary outcome measure was tumour regression grade in the primary tumour and LNs. Secondary outcomes were overall and disease-free survival.

### FDG PET-CT protocol

Patients were assessed with FDG PET-CT before and after completion of neoadjuvant chemotherapy, prior to surgery. The majority of patients had FDG PET-CT performed on one of two GE Discovery 710 PET-CT systems (GE Healthcare) at the host institution. Patients were fasted for a minimum of 6 h. A standard PET acquisition minimum coverage from skull base to upper thighs was acquired post-injection of a 350-MBq + / − 10% and an uptake time of 60 min at 3 min per bed position. The standard clinical attenuation–corrected PET reconstruction was a time-of-flight ordered subset expected maximisation reconstruction using 2 iterations, 24 subsets and a 6.4-mm Gaussian filter (VPFX, GE Healthcare) and thickness of 3.27 mm. The low-dose unenhanced CT scan was performed in shallow respiration, using a standardised protocol: 140 kV, pitch 1.375, 40 mm beam collimation (64 × 0.625 mm), 0.5-s rotation time and auto mA (15–100 mA, noise index 40). CT images were reconstructed with a thickness of 2.5 mm. A minority of patients had a combination of FDG PET-CT examinations performed on different scanners at the same institution, or different scanners at different institutions. Image analysis was undertaken using Hybrid Viewer (Hermes Medical Solutions) by a board-certified radiologist (M.S.) with 10 years of PET-CT reporting and who provides specialist input to the upper gastro-intestinal multi-disciplinary team meeting.

### Tumour measurements

A freehand region of interest (ROI) was used to calculate the SUV_max_ in the primary tumour. For oesophageal tumours, metabolic tumour length was calculated by multiplying the number of axial images the primary tumour was visible by the section thickness of the PET images. For junctional tumours with a non-vertical orientation, a freehand measurement was made in the most appropriate orthogonal imaging plane. Axial fused PET-CT imaging, with its ability to demonstrate residual mural thickening, was used to assist measurement of SUV_max_ in the primary tumour on post-treatment imaging when tumoural uptake was visually indistinguishable from background physiological oesophago-gastric uptake.

### Metabolic tumour response (mTR)

Receiver operating characteristic (ROC) curve analysis was used to determine optimum SUV_max_ and metabolic tumour length thresholds for predicting pTR in the study cohort. Metabolic tumour response (mTR) was defined using PERCIST (ΔSUV_max_ 30%) and MUNICON (ΔSUV_max_ 35%) and based on results of ROC curve analysis. Patients with a complete metabolic response (CMR), i.e. tumour indistinguishable from background physiological oesophago-gastric uptake, or partial metabolic response (PMR), i.e. reduction in tumour SUV_max_ ≥ pre-defined thresholds detailed above, were classified as responders. Patients with stable metabolic disease (SMD), i.e. reduction or increase in tumour SUV_max_ < predefined thresholds, or progressive metabolic disease (PMD), i.e. increase in tumour SUV_max_ ≥ predefined thresholds, were classified as non-responders.

### Nodal measurements

Metabolic nodal (mN) stage was defined as nodes discrete from the primary tumour within a standard lymphadenectomy territory visible above background soft tissue uptake and categorised as mN0 (0 avid nodes), mN1 (1–2 avid nodes), mN2 (3–6 avid nodes) or mN3 (> 6 avid nodes). A freehand ROI was drawn around the most avid node to determine nodal SUV_max_. Post-treatment nodal SUV_max_ measurement was not possible if nodal uptake was visually indiscernible from background soft tissue uptake and the size of the nodal residuum on axial fused PET-CT imaging was small thereby precluding reliable assessment.

### Metabolic nodal response

Metabolic nodal response (mNR) was defined as CMR (no discernible uptake above background soft tissue uptake), PMR (reduction in mN category or SUV_max_ ≥ 30%), SMD (stable mN category or reduction/progression in SUV_max_ < 30%) or PMD (progression of mN category or SUV_max_ ≥ 30%). ROC curve analysis was used to identify optimum SUV_max_ nodal threshold for predicting pNR in the study cohort.

### Pathological tumour and nodal response (pTR and pNR)

Pathological response was evaluated using the Mandard classification, a 5-point categorical scoring system based on the proportion of fibrosis and viable tumour cells in the surgical resection specimen [14]. Mandard scores were calculated for response in the primary tumour (pTR) and resected LN (pNR) as described previously [6]. Patients with Mandard scores of 1–3 were classified as responders and those with scores of 4–5 as non-responders.

### Treatment

All patients were on a curative-intent treatment pathway and received neoadjuvant chemotherapy followed by surgical resection. Patients who received extended neoadjuvant therapy as part of an advanced disease protocol were excluded. Transthoracic or transhiatal oesophagectomy or extended total gastrectomy was performed by either open or minimally invasive approach.

### Statistical analysis

Clinicopathological characteristics were compared using the chi-squared test. Overall survival was defined as time from surgery to death or date of last outpatient department visit. Disease-free survival was defined as time from surgery to cancer recurrence, death or date of last outpatient department visit. Continuous variables (SUV_max_ and metabolic tumour length) were evaluated using ROC analysis to identify the threshold which provided the greatest area under the curve (AUC) for predicting pathological response in the study cohort. An AUC of 1.0 indicated perfect predictive ability and an AUC of 0.5, no ability. Crude and adjusted logistic regression analyses were performed adjusting for age (continuous), sex (male or female), chemotherapy regimen (epirubicin, cisplatin and capecitabine (ECX) or fluorouracil, leucovorin, oxaliplatin and docetaxel (FLOT)) and presence of signet ring cells (yes or no). Survival curves were created using the Kaplan–Meier method, with subgroups compared using the log-rank test. Crude and adjusted survival analyses were performed using Cox proportional hazards regression adjusted for age (continuous), sex (male or female), chemotherapy regimen (ECX or FLOT), cT stage (cT1–2 or cT3–4), cN stage (cN0 or cN +), tumour grade (well/moderately differentiated or poorly differentiated) and presence of signet ring cells (yes or no). Confounders for logistic regression and Cox proportional hazards analyses were defined based on directed acyclic graphs [15]. Harrell’s C-index was calculated to compare the accuracy of different metabolic response parameters to predict survival. *p* values < 0.05 were considered to be statistically significant. Statistical analysis was performed using IBM SPSS statistics (IBM Corp, IBM SPSS statistics Version 27.0.) and GraphPad Prism 9 (GraphPad Software Inc.).

## Results

### Patient and treatment characteristics

There were 76 eligible patients identified from the institutional database. One patient had a non-avid tumour on baseline FDG PET-CT and was excluded, leaving 75 patients for final analysis. Mean age was 63 years (range 26–79) with the majority male (65/75, 86.7%) (Table 1). Almost two thirds received FLOT (48/75, 64.0%) with the remainder receiving ECX chemotherapy. Most patients (71/75, 94.7%) completed all prescribed cycles of neoadjuvant chemotherapy with a minority completing 2 cycles of ECX (*n* = 2) or 2 cycles of FLOT (*n* = 2). There was discordance between pathological response in the primary tumour and LN in several patients, including a significant proportion (6/26, 23.1%) who demonstrated a LN response in the absence of a response in the primary tumour. Median SUV_max_ pre- and post-chemotherapy, respectively, was 11.5 (range 3.8–47.6) and 4.4 (2.7–30.3) in the primary tumour and 4.4 (1.5–19.3) and 2.2 (1.0–8.2) in LNs. The majority of patients had baseline and post-treatment FDG PET-CT performed on the same scanner at the host institution (61/75, 81.3%). The PET scanners used for the remaining 14 patients are shown in Supplementary table 1. Stratified analysis excluding patients with FDG PET-CT performed on different scanners (Supplementary table 2, Supplementary table 3) did not demonstrate any major differences to clinicopathological, response or survival results, so this cohort was included in the final analysis.

**Table 1:** Clinicopathological and treatment characteristics of 75 patients with adenocarcinoma of the oesophagus or oesophago-gastric junction treated with neoadjuvant chemotherapy followed by surgical resection

Variable		Number (%)
Age at operation (mean)		63 years
Sex		
Male		65 (86.7%)
Female		10 (13.3%)
Tumour location		
Oesophagus		11 (14.7%)
Oesophago-gastric junction	Siewert type 1	11 (14.7%)
	Siewert type 2	45 (60.0%)
	Siewert type 3	8 (10.7%)
Operation		
Transthoracic oesophagectomy		50 (66.7%)
Transhiatal oesophagectomy		12 (16.0%)
Extended total gastrectomy		13 (17.3%)
Chemotherapy regimen		
ECX		27 (36.0%)
FLOT		48 (64.0%)
cT stage		
cT0–2		4 (5.3%)
cT3–4		71 (94.7%)
cN stage		
cN0		9 (12.0%)
cN1–3		66 (88.0%)
ypT stage		
ypT0–2		37 (49.3%)
ypT3–4		38 (50.7%)
ypN stage		
ypN0		39 (52.0%)
ypN1–3		36 (48.0%)
Tumour grade		
Well/moderate differentiation		39 (52.0%)
Poor differentiation		36 (48.0%)
Lymphovascular invasion		
No		43 (57.3%)
Yes		32 (42.7%)
Signet ring cell		
No		66 (88.0%)
Yes		9 (12.0%)
Resection margin		
R0		54 (72.0%)
R1		21 (28.0%)
Baseline and restaging FDG PET-CT performed on same scanner		
Yes		61 (81.3%)
No		14 (18.7%)
Pathological primary tumour regression (pTR)		
Responder (Mandard 1–3)		49 (65.3%)
Non-responder (Mandard 4–5)		26 (34.7%)
Pathological nodal regression (pNR)		
Responder (Mandard 1–3)		27 (36.0%)
Non-responder (Mandard 4–5)		21 (28.0%)
Negative nodes		24 (32.0%)
Not recorded		3 (4.0%)
Discordance between pTR and pNR		
pT responder and pN non-responder		5 (6.9%)
pN responder and pT non-responder		6 (8.3%)
mT responder and mN non-responder		3 (4.0%)
mN responder and mT non-responder		3 (4.0%)
Recurrence		
No recurrence		51 (68.0%)
Any recurrence		24 (32.0%)
Death		
Alive at end of follow-up		54 (72.0%)
Not alive at end of follow-up		21 (28.0%)
Median SUV_max_ (range)		
Tumour pre-chemotherapy		11.5 (3.8–47.6)
Tumour post-chemotherapy		4.4 (2.7–30.3)
Lymph node pre-chemotherapy		4.4 (1.5–19.3)
Lymph node post-chemotherapy		2.2 (1.0–8.2)

### Prognostic ability of metabolic tumour response to predict pathological tumour response

**Fig. 1:**
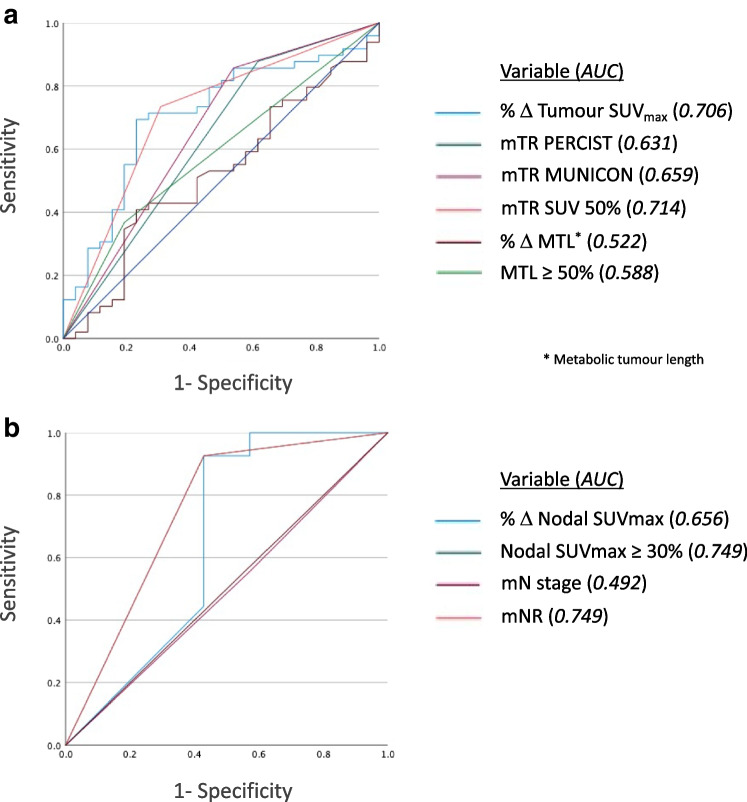
Receiver operating characteristic (ROC) analysis evaluating the ability of metabolic tumour response (mTR) parameters to predict pathological tumour response (**a**) and the ability of metabolic nodal parameters (mNR) to predict pathological nodal response or pathological node negativity in patients with metabolic positive nodes on baseline FDG PET-CT (**b**) in 75 patients with adenocarcinoma of the oesophagus or oesophago-gastric junction treated with neoadjuvant chemotherapy followed by surgical resection

ROC analysis (Fig. 1) demonstrated an optimum tumour SUV_max_ decrease of 51.2% for predicting pTR. Using a pragmatic cut-off of 50% (SUV 50%), this provided the best predictor of pTR (Table 2) (AUC 0.714, sensitivity 73.5%, specificity 69.2%, positive predictive value (PPV) 81.3%, negative predictive value (NPV) 58.1%, *p* < 0.001). This was more accurate than PERCIST (AUC 0.631, sensitivity 87.8%, specificity 38.5%, PPV 72.9%, NPV 62.5%, *p* = 0.008) and MUNICON (AUC 0.659, sensitivity 85.7%, specificity 46.2%, PPV 75.0%, NPV 63.2%, *p* = 0.003) which demonstrated higher sensitivity but significantly lower specificity. ROC analysis demonstrated an optimum reduction of metabolic tumour length of 46.9% for predicting pTR. Using a similarly pragmatic cut-off of 50%, this was less accurate than SUV_max_ (AUC 0.588, sensitivity 36.7%, specificity 80.8%, PPV 78.3%, NPV 40.4%, *p* = 0.118). mTR using SUV 50% and MUNICON provided the best concordance with pTR (*n* = 54, 72.0%), better than for PERCIST (*n* = 53, 70.7%) and reduction in metabolic tumour length (*n* = 39, 52.0%).

**Table 2:** Prognostic ability of metabolic response parameters to predict pathological response in 75 patients with adenocarcinoma of the oesophagus or oesophago-gastric junction treated with neoadjuvant chemotherapy followed by surgical resection

		Pathologic response							
		Responder^a^	Non-responder^b^	AUC^c^	Sensitivity	Specificity	PPV^d^	NPV^e^	*p* value
mTR^f^ PERCIST	Responder^g^	43 (72.9%)	16 (27.1%)	0.631	87.8%	38.5%	72.9%	62.5%	*p* = *0.008*
	Non-responder^h^	6 (37.5%)	10 (62.5%)						
mTR MUNICON	Responder^g^	42 (75.0%)	14 (25.0%)	0.659	85.7%	46.2%	75.0%	63.2%	*p* = *0.003*
	Non-responder^e^	7 (36.8%)	12 (63.2%)						
mTR SUV 50%	Responder^g^	36 (81.3%)	8 (18.2%)	0.714	73.5%	69.2%	81.3%	58.1%	*p* < *0.001*
	Non-responder^h^	13 (41.9%)	18 (58.1%)						
MTL^i^	Responder^j^	18 (78.3%)	5 (21.7%)	0.588	36.7%	80.8%	78.3%	40.4%	*p* = 0.12
	Non-responder^k^	31 (59.6%)	21 (40.4%)						
mNR^l^	mN responder^m^	25 (86.2%)	4 (13.8%)	0.749	92.6%	57.1%	86.2%	66.7%	*p* = *0.010*
	mN non-responder^n^	2 (33.3%)	4 (66.7%)						

Italics denote a statistically significant *p*-value

^a^Mandard 1–3 (includes pN-negative nodes for nodal response)

^b^Mandard 4–5

^c^Area under the curve

^d^Positive predictive value

^e^Negative predictive value

^f^Metabolic tumour response

^g^Complete/partial metabolic response

^h^Stable/progressive metabolic disease

^i^Metabolic tumour length

^j^ ≥ 50% decrease

^k^ < 50% decrease or increase

^l^Metabolic nodal response, excluding metabolic negative nodes on baseline and restaging FDG PET-CT

^m^Complete/partial metabolic response or decrease in mN stage

^n^Stable/progressive metabolic disease or stable/increase in mN stage

Logistic regression analysis (Table 3) demonstrated that all metabolic response criteria predicted pathological response except change in metabolic tumour length. However, SUV 50% was the most prognostic (SUV 50% odds ratio [OR] 7.41, 95% CI 2.23–24.61; MUNICON OR 4.87, 95% CI 1.37–17.30; PERCIST OR 4.05, 95% CI 1.09–15.04; change in tumour length OR 2.48, 95% CI 0.73–8.36). No other confounders in the adjusted model were predictive for pathological response. A further iteration of the model adjusting additionally for FDG PET-CT performed on separate scanners yielded similar results (SUV 50%, OR 7.33, 95% CI 2.20–24.47). Evaluation of metabolic response stratified by Mandard score in the primary tumour (Supplementary table 4) demonstrated that patients with a lower Mandard score (1–3) were more likely to be metabolic responders and also demonstrated improved survival.

**Table 3:** Logistic regression models evaluating the ability of metabolic tumour response (mTR) parameters and metabolic nodal response (mNR) to predict pathological response in 75 patients with adenocarcinoma of the oesophagus or oesophago-gastric junction treated with neoadjuvant chemotherapy followed by surgical resection, providing odds ratios (OR) and 95% confidence intervals (CI)

		Crude	Adjusted^a^
		OR (95% CI)	OR (95% CI)
MTL^b,c^	Non-responder^e^	1.00	1.00
	Responder^f^	**2.44** (0.78–7.59)	**2.48** (0.73–8.36)
mTR PERCIST^c^	Non-responder^g^	1.00	1.00
	Responder^h^	**4.48** (1.40–14.34)	**4.05** (1.09–15.04)
mTR MUNICON^c^	Non-responder^g^	1.00	1.00
	Responder^h^	**5.14** (1.69–15.62)	**4.87** (1.37–17.30)
mTR SUV 50%^c^	Non-responder^g^	1.00	1.00
	Responder^h^	**6.23** (2.19–17.75)	**7.41** (2.23–24.61)
mNR^d^	Non-responder^i^	1.00	1.00
	Responder^j^	**16.67** (2.09–133.05)	**17.19** (1.92–154.16)
	mN negative	**3.43** (0.56–21.18)	**3.31**(0.50–21.84)

Bold entries indicate statistically significant results

^a^Adjusted for: age, sex, chemotherapy regimen (FLOT/ECX), signet ring cell (yes, no)

^b^Metabolic tumour length

^c^Model evaluating pathological tumour response (Mandard 1–3)

^d^Model evaluating pathologic nodal response (Mandard 1–3) or node negativity

^e^ ≥ 50% decrease

^f^ < 50% decrease or increase

^g^Complete/partial metabolic response

^h^Stable/progressive metabolic disease

^i^Stable/progressive metabolic disease or stable/increase in mN stage

^j^Complete/partial metabolic response or decrease in mN stage

### Prognostic ability of metabolic nodal response to predict pathological nodal response

Analysis of metabolic nodal parameters (Table 4) demonstrated that percentage change of nodal SUV_max_ was more accurate than mN stage for predicting pNR or pathological node negativity (pN −) (SUV_max_ concordant *n* = 41, 56.9%; mN stage concordant *n* = 33, 45.8%). The combined category of mNR was equally as accurate as percentage change in nodal SUV_max_ alone.

**Table 4:** Prognostic ability of metabolic response parameters in lymph nodes and metabolic nodal response (mNR) to predict pathological nodal response (pNR) in 72 patients with adenocarcinoma of the oesophagus or oesophago-gastric junction treated with neoadjuvant chemotherapy followed by surgical resection

		pNR			
		pN responder^a^	pN non-responder^b^	pN negative	*p* value
Δ Nodal SUV_max_	Metabolic responder^c^	19 (67.9%)	3 (10.7%)	6 (21.4%)	*p* < *0.001*
	Metabolic non-responder^d^	2 (33.3%)	4 (66.7%)	0 (0%)	
	mN negative	6 (15.8%)	14 (36.8%)	18 (47.4%)	
mN stage	Metabolic responder^e^	11 (61.1%)	3 (16.7%)	4 (22.2%)	*p* = *0.002*
	Metabolic non-responder^f^	10 (62.5%)	4 (25.0%)	2 (12.5%)	
	mN negative	6 (15.8%)	14 (36.8%)	18 (47.4%)	
mNR	mN responder^g^	19 (67.9%)	3 (10.7%)	6 (21.4%)	*p* < *0.001*
	mN non-responder^h^	2 (33.3%)	4 (66.7%)	0 (0%)	
	mN negative	6 (15.8%)	14 (36.8%)	18 (47.4%)	

Italics denote a statistically significant *p*-value

^a^Mandard 1–3

^b^Mandard 4–5

^c^Complete/partial metabolic response

^d^Stable/progressive metabolic disease

^e^Decrease in mN stage

^f^Stable/increase in mN stage

^g^Complete/partial metabolic response or decrease in mN stage

^h^Stable/progressive metabolic disease or stable/increase in mN stage

ROC analysis (Fig. 1) excluding patients with metabolically negative nodes (mN −) at baseline (*n* = 38) demonstrated an optimum nodal SUV_max_ decrease of 32.6% for predicting pNR or pathological node negativity. Using an SUV_max_ threshold of 30% to define mNR (Table 2), this demonstrated high sensitivity and PPV but relatively low specificity and NPV for predicting pNR or pN − (AUC 0.749, sensitivity 92.6%, specificity 57.1%, PPV 86.2%, NPV 66.7%, *p* = 0.010). There was significantly higher concordance when excluding mN − patients compared with the entire cohort (85.3% vs 56.9%). Three patients had missing pNR data so were excluded from nodal analysis.

### Prognostic effect of pathological response on survival

**Fig. 2:**
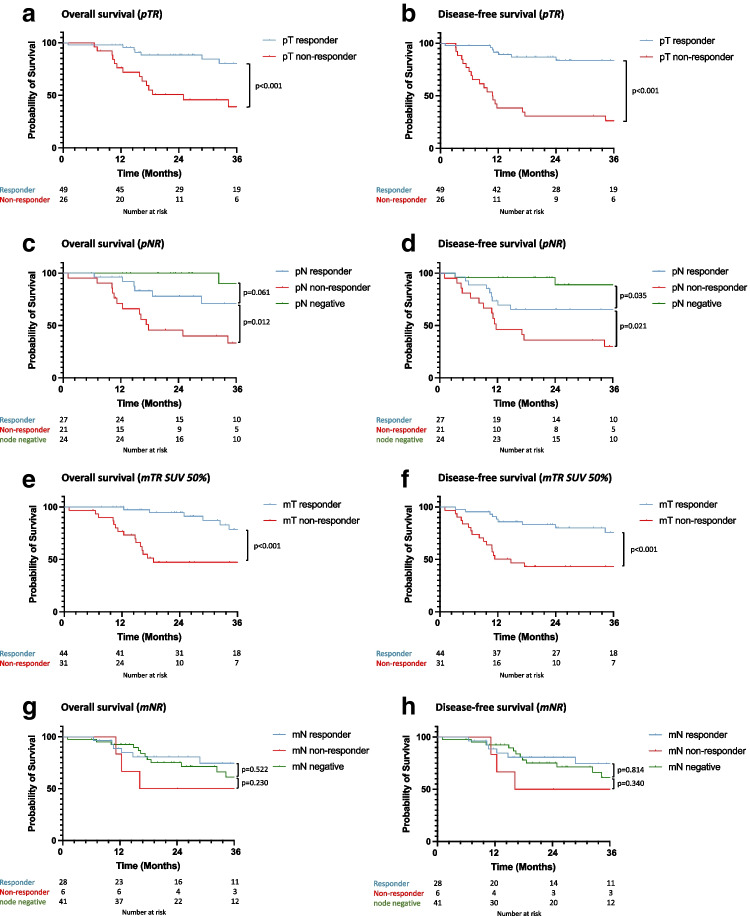
Kaplan–Meier survival analysis for overall and disease-free survival by pathological tumour response (pTR) (**a** + **b**), pathological nodal response (pNR) (**c** + **d**), metabolic tumour response (mTR) (**e** + **f**) and metabolic nodal response (mNR) (**g** + **h**) in 75 patients with adenocarcinoma of the oesophagus or oesophago-gastric junction treated with neoadjuvant chemotherapy followed by surgical resection

Kaplan–Meier (Fig. 2) and Cox regression survival analysis (Table 5) demonstrated better overall and disease-free survival in primary tumour pathological responders compared with non-responders (responder overall survival HR 0.10, 95% CI 0.03–0.34; disease-free survival HR 0.05, 95% CI 0.02–0.15). Pathological LN responders and patients with pathologically negative nodes had better survival than LN non-responders (responder overall survival HR 0.17, 95% CI 0.06–0.54; disease-free survival HR 0.28 (0.11–0.70); node negative overall survival HR 0.03, 95% CI 0.01–0.25; disease-free survival 0.05, 95% CI 0.01–0.26).

**Table 5:** Hazard ratios (HR) with 95% confidence intervals (CI) of overall and disease-free survival for metabolic tumour response (mTR), pathological tumour response (pTR), metabolic nodal response (mNR) and pathological nodal response (pNR) in 75 patients with adenocarcinoma of the oesophagus or oesophago-gastric junction treated with neoadjuvant chemotherapy followed by surgical resection. The accuracy of each mTR classification to predict survival was compared using Harrell’s C-index

		Overall survival		Disease-free survival	
		Crude	Adjusted^a^	Crude	Adjusted^a^
		HR (95% CI)	HR (95% CI)	HR (95% CI)	HR (95% CI)
mTR PERCIST	Non-responder^b^	1.00	1.00	1.00	1.00
	Responder^c^	0.45 (0.18–1.12)	0.50 (0.17–1.45)	0.39 (0.18–0.85)	0.41 (0.18–0.97)
	C-index	0.584	0.764	0.586	0.728
mTR MUNICON	Non-responder^b^	1.00	1.00	1.00	1.00
	Responder^c^	0.35 (0.15–0.82)	0.30 (0.10–0.84)	0.33 (0.16–0.71)	0.31 (0.13–0.73)
	C-index	0.639	0.788	0.621	0.738
mTR SUV 50%	Non-responder^b^	1.00	1.00	1.00	1.00
	Responder^c^	0.19 (0.08–0.50)	0.17 (0.06–0.48)	0.25 (0.11–0.55)	0.24 (0.10–0.58)
	C-index	0.732	0.831	0.679	0.773
pTR	Non-responder^d^	1.00 (reference)	1.00 (reference)	1.00 (reference)	1.00 (reference)
	Responder^e^	0.22 (0.09–0.54)	0.10 (0.03–0.34)	0.12 (0.05–0.29)	0.05 (0.02–0.15)
mNR	Non-responder^f^	1.00	1.00	1.00	1.00
	Responder^g^	0.40 (0.10–1.61)	0.16 (0.03–0.69)	0.57 (0.16–2.13)	0.19 (0.04–0.90)
	Node negative	0.56 (0.15–2.00)	0.30 (0.08–1.21)	0.64 (0.18–2.24)	0.32 (0.08–1.32)
pNR	Non-responder^d^	1.00	1.00	1.00	1.00
	Responder^e^	0.31 (0.12–0.82)	0.17 (0.06–0.54)	0.39 (0.17–0.89)	0.28 (0.11–0.70)
	Node negative	0.05 (0.01–0.39)	0.03 (0.01–0.25)	0.09 (0.02–0.37)	0.05 (0.01–0.26)

^a^Adjusted for: age, sex, chemotherapy regimen (FLOT/ECX), cT stage (cT0–2, cT3–4), cN stage (cN0, cN +), differentiation (well-mod, poor), signet ring cell (yes, no)

^b^Stable/progressive metabolic disease

^c^Complete/partial metabolic response

^d^Mandard 4–5

^e^Mandard 1–3

^f^Stable/progressive metabolic disease or stable/increase in mN stage

^g^Complete/partial metabolic response or decrease in mN stage

### Prognostic effect of metabolic response on survival

**Fig. 3:**
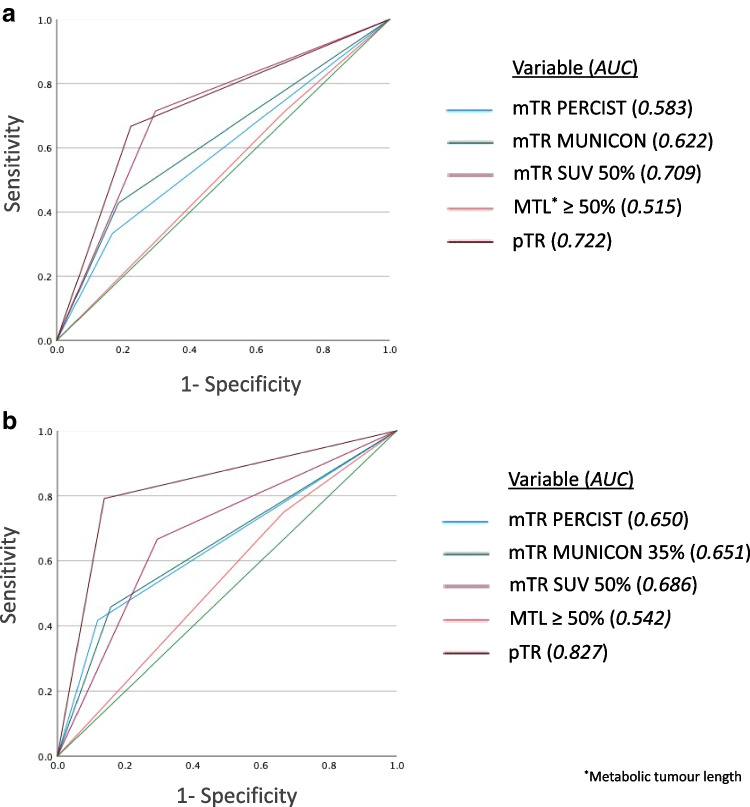
Receiver operating characteristic (ROC) analysis evaluating the prognostic effect of metabolic tumour response (mTR) parameters and pathological tumour response (pTR) on survival (**a**) and recurrence (**b**) in 75 patients with adenocarcinoma of the oesophagus or oesophago-gastric junction treated with neoadjuvant chemotherapy followed by surgical resection

Metabolic responders in the primary tumour demonstrated better overall and disease-free survival compared with non-responders using all SUV_max_ thresholds of mTR. However, when survival models were compared using Harrell’s C-index (Table 5), SUV 50% demonstrated the best predictive ability for overall and disease-free survival in both crude and adjusted analyses compared with PERCIST and MUNICON criteria. ROC analysis for metabolic and pathological tumour characteristics (Fig. 3) demonstrated pTR to be the best predictor of recurrence and survival, and SUV 50% was the second best predictor.

Metabolic nodal responders demonstrated improved survival compared with non-responders on adjusted analysis (responder overall survival HR 0.13, 95% CI 0.02–0.66; disease-free survival HR 0.19, 95% CI 0.04–0.90; node negative overall survival HR 0.30, 95% CI 0.08–1.21; disease-free survival 0.32, 95% CI 0.08–1.32). No other confounders in the adjusted model were prognostic for survival.

## Discussion

In this study, metabolic response on FDG PET-CT after neoadjuvant chemotherapy was predictive of pathological response in both the primary tumour and LN metastases of patients with adenocarcinomas of the oesophagus or oesophago-gastric junction. Patients who had a favourable metabolic or pathological response in the primary tumour or LNs had improved survival compared to non-responders.

Some methodological issues deserve attention. This study allowed for the long-term follow-up of a consecutive cohort of patients treated for oesophago-gastric adenocarcinoma and compared FDG PET-CT response with pathological LN response, making these results novel. Although most data were collected prospectively, the observational design made it difficult to exclude confounding. However, the results were adjusted for the key prognostic factors to counteract this. As a single-centre study, one advantage was that there was consistency of pathological and radiological reporting. This reduced heterogeneity; however, the single-centre design reduced the generalisability of the findings compared to a population-based approach. Although most patients had FDG PET-CT performed on the same scanner, the inclusion of 14 patients who did not may have introduced bias due to potential differences in SUV_max_ measurements and calibration between scanners. However, stratified analysis revealed no obvious differences in clinicopathological, response or survival results between these patients.

It has been suggested that commonly utilised thresholds of SUV_max_ are not optimal for assessing response to neoadjuvant therapy in oesophageal cancer, with higher cut-offs proposed [5, 16, 17]. This is supported by the results of the present study which suggest a reduction in SUV_max_ of 50% in the primary tumour was a better predictor of not only pathological response but also tumour recurrence and survival compared with PERCIST (ΔSUV_max_ 30%) and MUNICON (ΔSUV_max_ 35%). Although increasing the SUV_max_ threshold to 50% slightly decreased the sensitivity for predicting pathological response compared with PERCIST and MUNICON, it significantly increased the specificity and, overall, correctly identified pathological response in more patients. In this study, change in SUV_max_ was more accurate than change in metabolic tumour length for predicting pathological response and survival. However, advances in FDG PET-CT allowing calculation of three-dimensional spatial metrics such as metabolic tumour volume and total lesion glycolysis may provide even more reliable prognostic information [5]. Although such metrics were not calculated for the present study due to technical limitations, these results are still relevant since change in SUV_max_ remains the most reproducible and widely utilised metabolic parameter for response assessment.

The prognostic ability of FDG PET-CT to identify pathologic nodal disease was relatively poor. Approximately half of patients with metabolic negative nodes on baseline and restaging FDG PET-CT had pathological positive nodes, approximately a quarter of the patients in the entire cohort. This is not unexpected given that false-negative observations are well recognised, particularly with respect to small LNs due to a combination of limited tumour burden (insufficient to generate a detectable PET signal) and size below the spatial resolution of PET. When patients with metabolic negative nodes were excluded from response analysis to counteract the high false-negative rate, concordance, sensitivity and positive predictive value of mNR for predicting pNR or pathological node negativity were high, although specificity and negative predictive value remained low. Interestingly, there was also a false-positive rate of just over 10%, i.e. patients with metabolically positive but pathologically negative nodes, a finding which might be explained by post-treatment inflammation, also a well-recognised pitfall in FDG PET-CT interpretation.

The results of the present study support the findings of several previous studies demonstrating that a decrease in metabolic activity in the primary tumour following chemotherapy predicts pathological response [5, 18] and survival [3, 9, 19]. However, to our knowledge, only two studies from the same institution have evaluated the association between mNR and these outcomes [3, 5], with similar results obtained. Furthermore, no previous studies have evaluated the ability of FDG PET-CT to predict pathological nodal response. This is despite recent findings suggesting it is an independent predictor of survival and evidence of a discrepancy, 23% in this study, between patients who demonstrate a nodal response in the absence of a response in the primary tumour [6, 7]. The use of FDG PET-CT in assessing response to neoadjuvant chemotherapy in patients with oesophageal or junctional adenocarcinoma remains an under-researched area, and a large-scale study is needed to establish optimum response thresholds and its prognostic effect on pathological response and survival. Although this analysis focused on FDG PET-CT response after completion of neoadjuvant chemotherapy, at which point treatment alteration would not generally be considered, recent studies have evaluated early response assessment after induction chemotherapy in patients undergoing chemoradiation, with promising results [20]. Further research is warranted into whether FDG PET-CT performed earlier in the treatment pathway could be used to tailor individual treatment strategies in patients treated with neoadjuvant chemotherapy.

In conclusion, this study has demonstrated that metabolic response on FDG PET-CT was predictive of pathological response in both the primary tumour and LNs in a cohort of patients with locally advanced adenocarcinoma of the oesophagus or oesophago-gastric junction treated with neoadjuvant chemotherapy followed by surgical resection. However, a relatively high false-negative rate makes metabolic evaluation of pathological nodal response challenging. Metabolic tumour response, pathological tumour response, metabolic nodal response and pathological nodal response were all independent prognostic factors for survival.

## Supplementary Information

Below is the link to the electronic supplementary material.

Supplementary file1 (PDF 196 KB)

## Abbreviations

AUC: Area under the curve
CI: Confidence interval
CMR: Complete metabolic response
ECX: Epirubicin, cisplatin and capecitabine
FDG: 2-Deoxy-2[^18^F]fluoro-d-glucose
FLOT: Fluorouracil, leucovorin, oxaliplatin and docetaxel
HR: Hazard ratio
LN: Lymph node
mN stage: Metabolic nodal stage
mNR: Metabolic nodal response
mTR: Metabolic tumour response
NPV: Negative predictive value
OR: Odds ratio
PERCIST: PET Response Criteria in Solid Tumours
PET-CT: Positron emission tomography-computed tomography
PMD: Progressive metabolic disease
PMR: Partial metabolic response
pNR: Pathological nodal response
PPV: Positive predictive value
pTR: Pathological tumour response
ROC: Receiver operating characteristic
ROI: Region of interest
SMD: Stable metabolic disease
SUV_max_: Maximum standardised uptake value
TNM: Tumour, nodes and metastases

## Declarations

### Guarantor

The scientific guarantor of this publication is Mr. Andrew Davies.

### Statistics and biometry

Dr. Aida Santaolalla and Professor Mieke Van Hemelrijck (King’s College London) kindly provided statistical advice for this manuscript.

### Methodology

• Prospective

• observational

• performed at one institution
