# Supplementary material for: Metabolic tumour and nodal response to neoadjuvant chemotherapy on FDG PET-CT as a predictor of pathological response and survival in patients with oesophageal adenocarcinoma
Source: Eur Radiol. 2023 Mar 15;33(5):3647–59. doi: 10.1007/s00330-023-09482-7 (PMC10121512; doi:10.1007/s00330-023-09482-7)
Supplement: Supplementary file 1 — Supplementary file1 (PDF 196 KB) [file 330_2023_9482_MOESM1_ESM.pdf]

Supplementary table 1. List of PET CT scanners used for 14 patients who did not have both pre- and post- treatment FDG PET CT performed on one of two GE Discovery 710 PET-CT systems at Guy's and St Thomas' NHS Foundation Trust (GSTT).

Patient No	**Pre-treatment**		**Post-treatment**	
	Institution	Scanner	Institution	Scanner
1	GSTT	Siemens Biograph mCT	GSTT	GE Discovery 710
2	GSTT	Siemens Biograph mCT	GSTT	GE Discovery 710
3	GSTT	GE Discovery 710	External	GE Discovery MI DR
4	External	Siemens Biograph 6	GSTT	Siemens Biograph mCT
5	External	GE Discovery 710	GSTT	GE Discovery 710
6	External	GE Discovery MI DR	External	GE Discovery MI DR
7	GSTT	Siemens Biograph mCT	GSTT	GE Discovery 710
8	External	GE Discovery MI DR	GSTT	GE Discovery 710
9	External	GE Discovery 710	External	GE Discovery MI DR
10	External	GE Discovery 710	GSTT	Siemens Biograph mCT
11	GSTT	Siemens Biograph mCT	GSTT	GE Discovery 710
12	GSTT	Siemens Biograph mCT	GSTT	GE Discovery 710
13	GSTT	Siemens Biograph mCT	GSTT	GE Discovery 710
14	GSTT	Siemens Biograph mCT	GSTT	GE Discovery 710

Supplementary table 2. Prognostic ability of metabolic response parameters in the primary tumour to predict pathologic tumour response excluding patients with FDG PET-CT examinations performed on different scanners at diagnosis and following neoadjuvant chemotherapy in 61 patients with adenocarcinoma of the oesophagus or oesophago-gastric junction.

		**Pathologic response**		
		Responder [a]	Non-responder [b]	*p-value*
**mTR [c] PERCIST**	Metabolic responder [d]	34 (70.8%)	14 (29.2%)	*p=0.11*
	Metabolic non-responder [e]	6 (46.2%)	7 (53.8%)	
**mTR MUNICON**	Metabolic responder [d]	34 (73.9%)	12 (26.1%)	*p=0.016*
	Metabolic non-responder [e]	6 (40.0%)	9 (60.0%)	
**mTR SUV 50%**	Metabolic responder [d]	28 (80.0%)	7 (20.0%)	*p=0.006*
	Metabolic non-responder [e]	12 (46.2%)	14 (53.8%)	
**MTL [f]**	Metabolic responder [g]	11 (78.6%)	3 (21.4%)	*p=0.34*
	Metabolic non-responder [h]	29 (61.7%)	18 (38.3%)	
**mNR [i]**	mN responder [j]	15 (71.4%)	6 (28.6%)	*p=0.15*
	mN non-responder [k]	2 (33.3%)	4 (66.7%)	

[a] Mandard 1-3 (includes pN negative nodes for nodal response)

[b] Mandard 4-5

[c] metabolic tumour response

[d] Complete/partial metabolic response

[e] Stable/progressive metabolic disease

[f] Metabolic tumour length

[g] ≥50% decrease

[h] <50% decrease or increase

[i] Metabolic nodal response, excluding metabolic negative nodes on baseline and restaging FDG PET-CT

[j] Complete/partial metabolic response or decrease in mN stage

[k] Stable/progressive metabolic disease or stable/increase in mN stage

Supplementary table 3. Prognostic ability of metabolic response parameters in lymph nodes and metabolic nodal response (mNR) to predict pathologic nodal response (pNR) excluding patients with FDG PET-CT examinations performed on different scanners at diagnosis and following neoadjuvant chemotherapy in 58 patients with adenocarcinoma of the oesophagus or oesophago-gastric junction.

		pNR			
		pN responder [a]	pN non-responder [b]	pN negative	*p-value*
**Δ Nodal SUV_{max}**	Metabolic responder [c]	12 (57.1%)	3 (14.3%)	6 (28.6%)	*p=0.002*
	Metabolic non-responder [d]	2 (33.3%)	4 (66.7%)	0 (0%)	
	mN negative	4 (12.9%)	10 (32.3%)	17 (54.8%)	
**mN stage**	Metabolic responder [e]	6 (46.2%)	3 (23.1%)	4 (30.8%)	*p=0.018*
	Metabolic non-responder [f]	8 (57.1%)	4 (28.6%)	2 (14.3%)	
	mN negative	4 (12.9%)	10 (32.3%)	17 (54.8%)	
**mNR**	mN responder [g]	12 (57.1%)	3 (14.3%)	6 (28.6%)	*p=0.002*
	mN non-responder [h]	2 (33.3%)	4 (66.7%)	0 (0%)	
	mN negative	4 (12.9%)	10 (32.3%)	17 (54.8%)	

[a] Mandard 1-3

[b] Mandard 4-5

[c] Complete/partial metabolic response

[d] Stable/progressive metabolic disease

[e] Decrease in mN stage

[f] Stable/increase in mN stage

[g] Complete/partial metabolic response or decrease in mN stage

[h] Stable/progressive metabolic disease or stable/increase in mN stage

Supplementary table 4. Survival status at the end of follow-up and metabolic tumour response (mTR SUV 50%) stratified by Mandard score for patients with adenocarcinoma of the oesophagus or oesophago-gastric junction.

	**Survival status**		*p-value*
	Alive	Not-alive	
**Mandard 1** [a]	8 (100%)	0	
**Mandard 2** [b]	10 (90.9%)	1 (9.1%)	
**Mandard 3** [c]	24 (80.0%)	6 (20.0%)	*p<0.001*
**Mandard 4** [d]	10 (50.0%)	10 (50.0%)	
**Mandard 5** [e]	2 (33.3%)	4 (66.7%)	
	**Metabolic tumour response**		*p-value*
	Responder	Non-responder	
**Mandard 1**	8 (100%)	0	
**Mandard 2**	9 (81.8%)	2 (18.2%)	
**Mandard 3**	19 (63.3%)	11 (36.7%)	*p=0.002*
**Mandard 4**	6 (30.0%)	14 (70.0%)	
**Mandard 5**	2 (33.3%)	4 (66.7%)	

[a] Complete response
[b] <10% residual tumour
[c] 10-50% residual tumour
[d] >50% residual tumour
[e] No regression
